# Dexmedetomidine Preconditioning Protects Rats from Renal Ischemia–Reperfusion Injury Accompanied with Biphasic Changes of Nuclear Factor-Kappa B Signaling

**DOI:** 10.1155/2020/3230490

**Published:** 2020-04-17

**Authors:** Naren Bao, Bing Tang, Junke Wang

**Affiliations:** Department of Anesthesiology, Hospital of China Medical University, 155 Nanjingbei Street, Heping District, Shenyang, Liaoning 110001, China

## Abstract

Acute kidney injury (AKI) is one of the most common and troublesome perioperative complications. Dexmedetomidine (DEX) is a potent *α*2-adrenoceptor (*α*2-AR) agonist with anti-inflammatory and renoprotective effects. In this study, a rat renal ischemia–reperfusion injury (IRI) model was induced. At 24 h after reperfusion, the IRI-induced damage and the renoprotection of DEX preconditioning were confirmed both biochemically and histologically. Changes in nuclear factor-kappa B (NF-*κ*B), as well as its downstream anti-inflammatory factor A20 and proinflammatory factor tumor necrosis factor-*α* (TNF-*α*), were detected. Atipamezole, a nonselective antagonist, was then added 5 min before the administration of DEX to further analyze DEX's effects on NF-*κ*B, and another anti-inflammatory medicine, methylprednisolone, was used in comparison with DEX, to further analyze DEX's effects on NF-*κ*B. Different concentrations of DEX (0 nM, 0.1 nM, 1 nM, 10 nM, 100 nM, 1 *μ*M, and 10 *μ*M) were applied to preincubated human renal tubular epithelial cell line (HK-2) cells in vitro. After anoxia and reoxygenation, the MTT (3-(4,5-dimethylthiazol-2-yl)-2,5-diphenyltetrazolium bromide) tetrazolium assay and enzyme-linked immunosorbent assay (ELISA) were performed to evaluate the levels of NF-*κ*B downstream anti-inflammatory cytokines. The results showed that, unlike methylprednisolone, DEX preconditioning led to a time-dependent biphasic change (first activation then inhibition) of NF-*κ*B in the rat renal IRI models that were given 25 *μ*g/kg i.p. It was accompanied by a similarly biphasic change of TNF-*α* and an early and persistent upregulation of A20. In vitro, DEX's cellular protection showed a concentration-dependent biphasic change which was protective within the range of 0 to 100 nM but became opposite when concentrations are greater than 1 *μ*M. The changes in the A20 and NF-*κ*B messenger RNA (mRNA) levels were consistent with the renoprotective ability of DEX. In other words, DEX preconditioning protected the rats from renal IRI via regulation biphasic change of NF-*κ*B signaling.

## 1. Introduction

Acute kidney injury (AKI) is one of the most common and troublesome complications in hospitalized patients after surgery, hemorrhagic shock, cardiac arrest, and sepsis [1, 2]. AKI is not merely single-organ dysfunction related to endothelial injury, cellular apoptosis, and oxidative stress; it also causes inflammatory reactions involving neutrophil migration and cytokine release. This can result in damage to the extrarenal organs, such as the lung, heart, liver, and brain, and it can often lead to significant increases in mortality, hospital stays, and medical-related costs [3–6]. According to the Kidney Disease: Improving Global Outcomes (KDIGO) definition, AKI leads to complications in 18.3% of all hospitalized patients and 57% of critically ill patients, with an associated in-hospital mortality of 11% and 27%, respectively [7, 8].

A major pathological process in AKI, kidney ischemia–reperfusion injury (IRI), is an inflammatory reaction in which the nuclear factor-kappa B (NF-*κ*B) family plays a key role [9–15]. Dexmedetomidine (DEX), a potent *α*2-adrenoceptor (*α*2-AR) agonist that is widely used perioperatively, has been recently found to have protective and anti-inflammatory effects on organs [16–20]. The mechanisms underlying DEX's renal protection could include the inhibition of NF-*κ*B and its downstream proinflammatory factor [21, 22]. Interestingly, *α*2-ARs are classic guanosine triphosphate (GTP) protein-coupled receptors (GPCR). Their agonists are coupled primarily with the inhibitory subunits (G_i_). This leads to the inhibition of downstream adenylate cyclase (AC), followed by the inhibition of cyclic adenosine monophosphate (cAMP), and, eventually, the activation of NF-*κ*B signaling [23–25]. By now, the specific connection between DEX and NF-*κ*B signaling has yet to be elucidated. And many researches on the possible protective or damaging effects of *α*2-AR agonism are inconclusive [26, 27]. It seems that there are multiple, and sometimes paradoxical, relationships among NF-*κ*B, *α*2-AR agonism, and organ protection.

In order to determine how *α*2-AR agonism participates in the changes of NF-*κ*B signaling while generating renal protective effects, we established a classic rat renal ischemia–reperfusion model to observe the fluctuations in NF-*κ*B signaling at different time points after IRI. Comparisons of the effects of methylprednisolone, an NF-*κ*B inhibitor and anti-inflammatory agent, facilitated the determination of possible correlations. Second, the study examined the effects of different doses (subclinical, clinical, or much larger doses) of DEX on the possible relationship to the changes in NF-*κ*B signaling and cell survival.

## 2. Materials and Methods

### 2.1. Animals

The experimental protocols were performed in accordance with the Guide for the Care and Use of Laboratory Animals of China Medical University. They were approved by the China Medical University Ethics Review Committee for Animal Experimentation (Shenyang, Liaoning, China). Healthy male 7–8-week-old Sprague-Dawley rats weighing 220–270 g were housed under pathogen-free conditions. The animals were acclimated to the laboratory conditions (22°C ± 1°C, 12–12 h light–dark cycle, and ad libitum access to food and water) for 1 week prior to the experiment, and they were fasted 24 h before the operation.

### 2.2. Renal Ischemia–Reperfusion Injury Model

Renal IRI was induced by right renal pedicle clamping for 45 min and left nephrectomy under 1.5% isoflurane surgical anesthesia. The successful restoration of blood perfusion was indicated by the change in the color of the kidney from dark purple to reddish brown within 5 min of the release of the arterial clip. After the incision was sutured, 0.2% ropivacaine was administered subcutaneously. The rectal temperatures were maintained at 37°C ± 1°C with a heating pad. The heart rates were monitored with subcutaneous electrodes. A 24 G trocar was placed in each left femoral artery to monitor invasive arterial pressure. The exclusionary criteria were a mean blood pressure (MAP) lower than 55 mmHg or a heart rate slower than 200 beats per min (bpm) for more than 5 min. Each animal received an i.p. injection of 0.5 mL saline every 2 h until it awoke or until the specimen was collected.

### 2.3. Experimental Protocol

**Figure 1:**
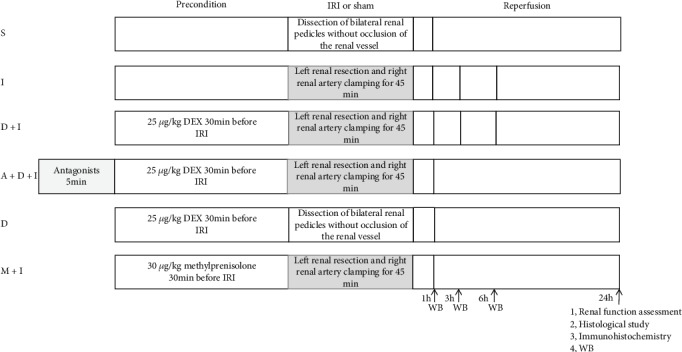
Experimental groups and protocol. Schematic diagram of the six groups of rats exposed to the experimental treatments.

The rats were randomly assigned to six groups (Figure 1). Group 1, the S (sham) group: bilateral renal pedicle dissection was performed without renal vessel occlusion. Group 2, the I (IRI) group: the rats underwent left renal resection and right renal artery clamping for 45 min. Group 3, the D+I (DEX preconditioning) group: the rats received 25 *μ*g/kg DEX i.p. (on the basis of previous studies [28]) 30 min before the establishment of renal IRI. Group 4, A+D+I (antagonist) group: the *α*2-AR antagonist atipamezole (250 *μ*g/kg) was injected i.p. 5 min before the administration of 25 *μ*g/kg DEX and the establishment of renal IRI 30 min later. Group 5, D (DEX) group: the rats received 25 *μ*g/kg DEX i.p., and 30 min later, they underwent the same operation as those in the S group. Group 6, M+I (methylprednisolone preconditioning) group: the rats received 30 mg/kg methylprednisolone i.p. 30 min before the establishment of renal IRI.

### 2.4. Renal Function Assessment

Twenty-four hours after reperfusion, five animals in each group (S, I, D+I, A+D+I, D, and M+I) underwent systemic heparinization (200 U/kg) and blood sample acquisition. In addition, the blood was centrifuged at 3,000 rpm for 10 min at 4°C. The supernatant was numbered and preserved at −70°C for the assessment of creatinine (Cr) and urea nitrogen (BUN) with a biochemical analyzer.

## 3. Histological Study

Five animals in each group were sacrificed 24 h after the reperfusion or sham operations. The kidney was collected from each rat and fixed in neutral formaldehyde solution for 24 h before being immersed in a phosphate buffered saline (PBS) solution. The tissues were embedded in paraffin, cut into 5 *μ*m sections, and stained with hematoxylin and eosin (HE) for light microscopy. Renal damage was evaluated against the modified criteria described by Paller et al. [29]. Five nonrepeating visualization fields were observed under ×400 magnification, and 10 renal tubules were scored in each field of view.

### 3.1. Nuclear Factor-Kappa B p65 Signaling Analysis by Western Blot

First, the NF-*κ*B p65 signaling in Groups I and D+I were compared at 1, 3, 6, and 24 h after IRI. This allowed for the time point at which the greatest changes in NF-*κ*B p65 signaling were observed to be the basis for the comparisons of the groups (S, I, D+I, A+D+I, D, and M+I). The rats were sacrificed at different time points.

The kidneys were homogenized, and the nuclear and cytoplasmic extracts from each specimen were purified through the use of nucleoprotein and cytoplasmic protein extraction kits (KGP-150; Kangchen, Shanghai, China). The manufacturer's instructions were followed. The samples were separated through sodium dodecyl sulfate polyacrylamide gel electrophoresis (SDS-PAGE) and electrotransferred onto a nitrocellulose membrane. Subsequently, the membrane was blocked with 5% nonfat milk at room temperature for 2 h. This was followed by overnight incubation with rabbit polyclonal anti-NF-*κ*B p65 antibody (1 : 500, 8242S, Cell Signaling Technology, USA), rabbit anti-TNF-*α* antibody (1 : 500, ab6671, Abcam, USA), rabbit polyclonal anti-A20/TNFAIP3 antibody (1 : 500, 5630S, Cell Signaling Technology, USA), or rabbit polyclonal anti-glyceraldehyde-3-phosphate dehydrogenase (GAPDH) antibody (1 : 500, Santa Cruz, USA). The membranes were then washed and incubated with a secondary antibody, goat anti-rabbit HRP-conjugated IgG (1 : 4,000, Cell Signaling Biotechnology, USA), for 2 h. The blotted protein bands were visualized with enhanced chemiluminescence (ECL) western blot detection reagents (Amersham, USA). The band density measurements were correlated with protein expression and normalized to the GAPDH band density.

### 3.2. Immunohistochemistry for Detection of Nuclear Factor-Kappa B p65 and A20

One hour after reperfusion or the sham operation, each rat was transcardially perfused with cold saline under deep anesthesia. The right renal pedicle was separated, and the kidney was removed after the kidney capsule was gently peeled off. The kidney was then cut to two parts. One part was fixed in 4% paraformaldehyde overnight, and the other was stored at −70°C for further western blot analysis. After gradient dehydration, the tissues were immersed in liquid paraffin and embedded prior to the preparation of 5 *μ*m thick paraffin sections. Next, the paraffin sections were dewaxed to water, rinsed with distilled water, and soaked in PBS for 5 min. To eliminate endogenous peroxidase activity, 3% hydrogen peroxide (H_2_O_2_) was incubated at room temperature for 5 to 10 min. The primary antibody, NF-*κ*B p65 (1 : 500, Cell Signaling Technology, USA, 8242S), and A20 (1 : 300, Abcam, ab13597) were added dropwise and incubated at 37°C for 1 to 2 h. Reagent 1 (polymer helper) was added, and this was followed by incubation at room temperature or 37°C for 20 min. Polyperoxidase-anti-rabbit IgG was added and incubated at room temperature or 37°C for 20 to 30 min.

After each of the aforementioned steps, the sections were rinsed with PBS three times for 2 min. The developer (diaminobezidine, DAB) was then added. The sections were fully rinsed with tap water, counterstained, dehydrated and sealed, and then photographed. The A20 protein, which is expressed mainly in the cytoplasm, appears as brownish yellow granules; NF-*κ*B p65 protein, which is expressed in the cytoplasm and/or nucleus, appears as brownish yellow granules.

### 3.3. In Vitro Experiments

#### 3.3.1. Cell Culture and Hypoxia Reoxygenation

Human renal proximal tubular epithelial cells (HK-2, Chinese Academy of Medical Sciences) were cultured and passaged every 3 to 4 days in 100 mm dishes containing RPMI-1640 medium supplemented with 10% fetal bovine serum, 100 U/mL penicillin, and 100 *μ*g/mL streptomycin (Sigma-Aldrich). The cells were incubated in a humidified atmosphere of 5% CO_2_ and 95% air at 37°C for 24 hours and subcultured at 70–80% confluence. For the experiments, the HK-2 cells were plated onto 60 mm dishes in a medium containing 10% fetal bovine serum for 24 h. They were then switched to RPMI-1640, a serum-free medium, for 24 hours. At the end of treatment, the cells were harvested for further analysis.

After overnight adhesion, the cells were treated with media containing DEX. To facilitate the detection of time-dependent effects, the cells were treated for 30 min. At the end of incubation, the drug-containing medium was replaced with 0.1 mL DEX-free RPMI-1640, and 0.5 mg/mL MTT was added to each well prior to 4 h of incubation.

### 3.4. Cell Viability

Cell viability was determined through an MTT assay. The HK-2 cells were plated into 96-well plates at a density of 5 × 10^3^ cells/well and incubated with 0.5 mg/mL MTT (Sigma-Aldrich, St. Louis, MO) for 3 h. They were then centrifuged at 1,800 rpm at room temperature for 10 min to remove the supernatant. Subsequently, formazan was extracted from the pelleted cells with 600 dL of dimethyl sulfoxide (DMSO) for 15 min. The optical density (OD) was measured on an enzyme-linked detector, and the detection wavelength was 490 nm. Each treatment was set up with 2–8 replicate wells with a blank control. Cell viability was calculated on the basis of the OD: cell survival rate (%) = (OD of hypoxic cultured cells − OD of blank control)/(OD of normal culture cells–OD of blank control).

### 3.5. Real-Time Reverse Transcription Polymerase Chain Reaction Detection of A20 and Nuclear Factor-Kappa B Messenger RNA

Reverse transcription polymerase chain reaction (RT-PCR) analysis of total RNA and then cDNA were prepared from cultured cells through the use of TRIzol reagent (Invitrogen Life technology, USA) and a PrimeScript™ RT reagent Kit (Takara, Japan). The following primers were used: human NF-*κ*B p65, upstream: 5′-CTTCCTGCCCTACAGAGGTC-3′, downstream: 5′-GAGCAGTCTGTTGCACTGGT-3′; human A20 (TNFAIP3), upstream: 5′-CACACAAGGACTTGGATC-3′, downstream: 5′-CTGTAGTCCTTTTGAAGCAAGTACTG-3′; and human GAPDH, upstream: 5′-CCAGGCGCCCAATACGACCAAA-3′, downstream: 5′-TTCTTTTGCGTCGCCAGCCGAG-3′. Real-time PCR was performed in a 96-well optical reaction plate; SYBR Premix Ex TaqT MII (Takara, Japan) was used. The real-time PCR reactions were performed on Agilent Mx3000P QPCR systems (Agilent, CA).

### 3.6. Selection of Hypoxia Duration

The HK-2 cells were placed in an incubator containing 5% CO_2_ and 1% O_2_ for 0, 1, 2, 3, or 6 h. The shortest hypoxic time for more than 50% of the cells that died was selected on the basis of the changes in cell activity (MTT assay) as a follow-up test condition.

### 3.7. Dexmedetomidine Concentrations for Human Renal Proximal Tubular Epithelial Cell Incubation

The HK-2 cells were treated with different DEX concentrations (0 nM, 0.1 nM, 1 nM, 10 nM, 100 nM, 1 *μ*M, or 10 *μ*M), and this was followed with hypoxia–reoxygenation. Cell activity A20 and NF-*κ*B p65 messenger RNA (mRNA) levels were observed.

### 3.8. Statistical Analysis

All statistical analyses were performed in IBM SPSS Statistics for Windows, Version 23.0 (IBM Corp., Armonk, NY, USA). The histological scores, which are presented as medians (interquartile ranges), were analyzed with the Kruskal–Wallis test. The quantitative data are presented as the means ± standard deviations (SDs). Multiple comparisons of the groups were analyzed through the use of one-way repeated-measures analysis of variance (ANOVA) with the Bonferroni post hoc tests. Differences were considered statistically significant at *p* < 0.05.

## 4. Results

### 4.1. Dexmedetomidine Preconditioning Provided Renoprotective Effects

At 24 h after reperfusion, the IRI group (Group I) exhibited obvious kidney damage, with an increase in the BUN levels (*p* < 0.01 vs. Group S). As is shown in Table 1, DEX preconditioning significantly mitigated the deterioration of BUN (*p* < 0.01 vs. Group I), and this could be completely reversed by atipamezole (*p* < 0.01 vs. Groups D+I and S; *p* > 0.05 vs. Group I). The methylprednisolone preconditioning yielded effects that were similar to but less protective than those observed for DEX (*p* < 0.01 vs. Group I; *p* < 0.05 vs. Group S). Similar results were observed for the Cr levels. The Group D values were normal (Table 1).

**Figure 2:**
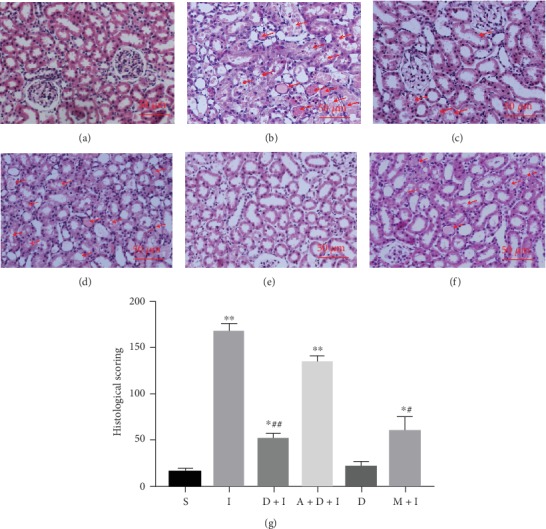
Histological tests showed that dexmedetomidine (DEX) preconditioning provided renoprotective effects. The rats underwent left renal resection and right renal artery clamping for 45 min (renal ischemia–reperfusion injury (IRI)) or just dissection of the bilateral renal pedicles without occlusion (sham). Either DEX or methylprednisolone was given 30 min before ischemia. Representative microphotographs were taken from the following groups: (a) sham, (b) IRI, (c) DEX (25 *μ*/kg)+IRI, (d) atipamezole (250 *μ*/kg)+DEX+IRI, (e) DEX alone, and (f) methylprednisolone (30 mg/kg)+IRI. (g) Quantification of the histological scoring following IRI in rats preconditioned as above. The histological damage was indicated by red arrows. Bar = 50 *μ*m. Data are mean ± standard deviation (SD; *n* = 5). ^∗^*p* < 0.05, ^∗∗^*p* < 0.01 vs. S; ^#^*p* < 0.05, ^##^*p* < 0.01 vs. I.

The histopathological assessment of tubular damage was conducted by an investigator who was blinded to the experimental protocol. The junction of the renal cortex and medulla was observed under a light microscope. IRI (Group I) caused significant pathological injury (*p* < 0.01 vs. Group S), including obvious renal tubule dilatation, swollen or flat tubular epithelial cells, and various types of degeneration, shedding, and necrosis of these cells (Figure 2). DEX (Group D+I) significantly reduced renal IRI. Microscopically, the overall renal tubular structure was relatively complete. There was tubular luminal dilatation and some granular or watery degeneration of the tubular epithelial cells. The nonselective *α*2-AR antagonist, atipamezole (Group A+D+I), significantly inhibited DEX's renal protective effects, which were expressed as the extensive degeneration of the epithelial cells and obvious exudates and casts in the renal tubules. DEX (Group D) did not cause changes in the renal histopathology. There was only a slight expansion of the tubular lumen. Methylprednisolone (Group M+I) was also found to provide good renal protection. There was no significant difference between these results and those for DEX.

### 4.2. Dexmedetomidine Preconditioning Led to a Time-Dependent Biphasic Change (First Activation Then Inhibition) of NF-*κ*B

**Figure 3:**
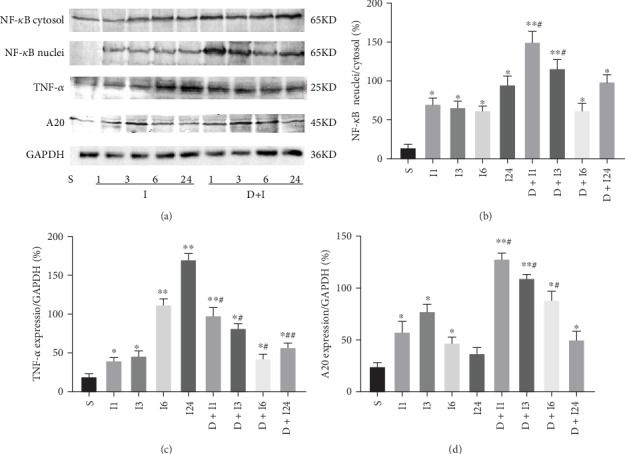
(a) Nuclear factor-kappa B (NF-*κ*B) signaling changes after kidney IRI and DEX preconditioning. (b) DEX preconditioning led to a time-dependent biphasic change of NF-*κ*B of the early activation (within 3 h of IRI) and later inhibition (6 h after IRI) of NF-*κ*B signaling in the rat renal IRI models that were given 25 *μ*g/kg i.p. (c) It was accompanied by the early (<3 h) upregulation and later (>6 h) significant inhibition of TNF-*α* and (d) the persistent upregulation of A20. The data are the mean ± standard deviation (SD; *n* = 5). ^∗^*p* < 0.05, ^∗∗^*p* < 0.01 vs. S; ^#^*p* < 0.05, ^##^*p* < 0.01 vs. I.

To determine the changes in the NF-*κ*B signaling underlying the renoprotective effects of DEX at different time points, renal tissues were collected from Groups I and D+I at 1, 3, 6, and 24 h after reperfusion (*n* = 5 per group) for western blot and compared with those from Group S. First, at each time point, NF-*κ*B nuclear transfer was found to be significantly higher in Groups I and D+I than in Group S (*p* < 0.05 vs. Group S). DEX led to a time-dependent biphasic change of NF-*κ*B that at the first activation (within 3 h especially at 1 h after IRI) then inhibition (1 h, *p* < 0.01; and 3 h, *p* < 0.05 vs. Group I) at 6 h and 24 h after reperfusion, the NF-*κ*B in Group D+I was similar to that in Group I (*p* > 0.05 vs. Group I; Figure 3).

### 4.3. Dexmedetomidine Preconditioning Continuously Increased A20 Expression and Temporarily Increased and Then Significantly Inhibited TNF-*α* Expression

The analysis of the NF-*κ*B downstream anti- and proinflammatory proteins (A20 and TNF-*α*) at the same time points after reperfusion indicated that IRI led an moderate increase in A20 which peaks at 3 h after reperfusion. DEX preconditioning advanced the peak to 1 h after reperfusion (*p* < 0.01 vs. Group S) and remained at a high level thereafter (*p* < 0.05 at each time point vs. Group S; Figure 3).

The results for the important proinflammatory cytokine TNF-*α* were even more interesting. First, the TNF-*α* levels in Groups I and D+I were significantly higher than those observed in Group S (*p* < 0.05 vs. Group S). However, Group D+I showed a biphasic change of temporarily upregulated (*p* < 0.05 vs. Group S) and then significantly downregulated (*p* < 0.05 vs. Group S) levels of TNF-*α* which was similar to the changes observed with NF-*κ*B. This suggested a return to the anti-inflammatory conditions that were consistent with the above-described renoprotective effects of DEX (at 6 h, *p* < 0.05; and at 24 h, *p* < 0.01 vs. Group I).

### 4.4. Comparisons of Nuclear Factor-Kappa B Signaling at 1 Hour after Reperfusion

**Figure 4:**
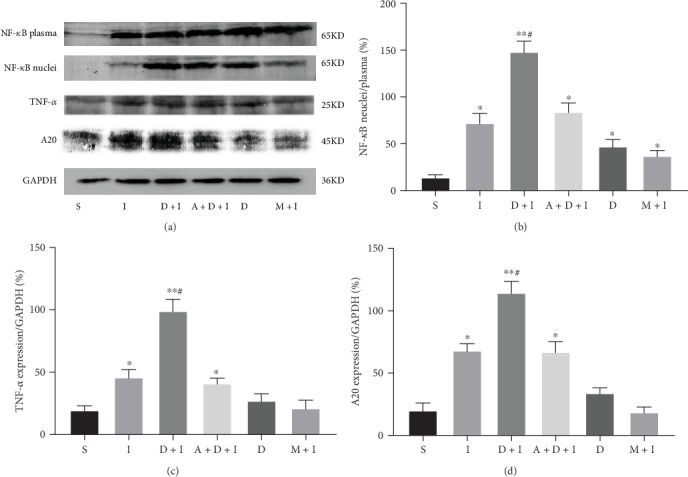
(a) Comparison of nuclear factor-kappa B (NF-*κ*B) signaling in the groups after 1 hour of reperfusion. Dexmedetomidine (DEX) preconditioning caused the significant activation of (b) NF-*κ*B and the upregulation of (c) tumor necrosis factor-*α* (TNF-*α*) and (d) A20 expression. This was completely reversed by atipamezole. Only DEX without IRI also increased the nuclear translocation of NF-*κ*B and mildly increased A20. The data are the mean ± standard deviation (SD; *n* = 5). ^∗^*p* < 0.05, ^∗∗^*p* < 0.01 vs. S; ^#^*p* < 0.05, ^##^*p* < 0.01 vs. I.

Because the most obvious changes in NF-*κ*B signaling were observed within 1 h of IRI, this time point was selected for the comparisons of the six groups (S, I, D+I, A+D+I, D, and M+I). DEX preconditioning was found to cause the significant activation of NF-*κ*B and the upregulation of A20 (*p* < 0.01 vs. Group S; and *p* < 0.05 vs. Group I, respectively). Atipamezole reversed this effect to levels similar to those in Group I. Most important, DEX treatment in the absence of IRI also increased the nuclear translocation of NF-*κ*B (*p* < 0.05 vs. Group S) and slightly increased the expression of A20 (*p* > 0.05 vs. Group S). The changes observed for TNF-*α* were similar to those observed for A20 (Figure 4).

**Figure 5:**
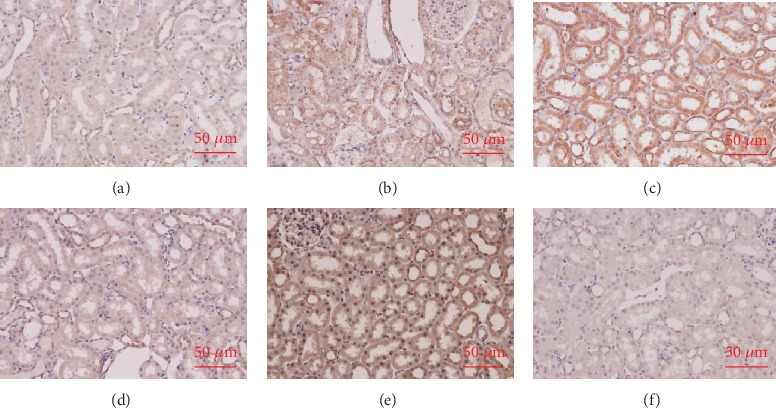
Dexmedetomidine (DEX) preconditioning activated nuclear factor-kappa B (NF-*κ*B) 1 hour after reperfusion. NF-*κ*B activation was assessed immunohistochemically. (a) The nuclear translocation of NF-*κ*B in Group S. (b) The nuclear translocation of NF-*κ*B was observed in the renal tubules of Group I. (c) DEX preconditioning (D+I group) significantly increased NF-*κ*B nuclear translocation. (d) These effects were obviously attenuated by atipamezole (A+D+I group). (e) DEX without IRI also induced a certain amount of NF-*κ*B activation. (f) Methylprednisolone, as an anti-inflammatory agent, obviously inhibited NF-*κ*B, thereby indicating that the underlying mechanism was different from that in DEX.

**Figure 6:**
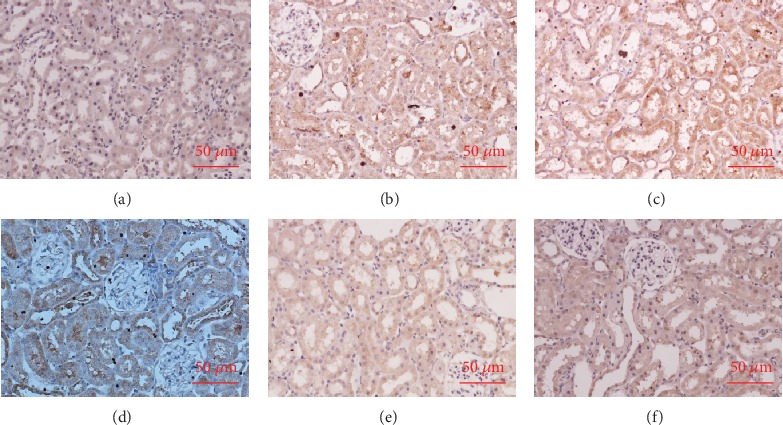
Dexmedetomidine (DEX) preconditioning upregulated A20 at 1 hour after reperfusion. A20 activation was assessed immunohistochemically. (a) The A20 expression in Group S. (b) The upregulation of A20 was observed in the renal tubules of Group I. (c) DEX preconditioning (D+I group) further increased the expression of A20. (d) Atipamezole (A+D+I group) reversed this effect. (e) DEX produced a slight increase in A20 expression over that observed in Group S (A). (f) Methylprednisolone obviously inhibited A20.

The immunohistochemistry results indicated that there were consistent changes in the NF-*κ*B and A20 in the renal tubular cells. The nuclear translocation of NF-*κ*B and a small amount of A20 expression in the cytoplasm were observed in the renal tubules of the Group I rats. DEX preconditioning (D+I group) significantly increased NF-*κ*B nuclear translocation and A20 upregulation. These effects were obviously mitigated by atipamezole (A+D+I group). DEX without IRI also induced a certain amount of NF-*κ*B activation and a slight increase in A20 expression. Methylprednisolone, as an anti-inflammatory agent, obviously inhibited NF-*κ*B and its downstream A20. This suggests that its underlying mechanism was different from that in DEX (Figures 5 and 6).

### 4.5. Dexmedetomidine Preconditioning Showed a Concentration-Dependent Biphasic Change in Cell Protection Which Was Positively Correlated with the Nuclear Factor-Kappa B and A20 Variations

As was previously explained, the in vivo changes in the NF-*κ*B signaling pathway, with or without the application of DEX preconditioning, were observed along a time axis. Next, the in vitro effects of the different DEX concentrations on the NF-*κ*B signaling pathway were observed.

First, the most appropriate hypoxia time for the experiments by MTT assay was determined. Cell viability decreased significantly to 37% ± 9.2% (>50%) after 3 h of hypoxia (*p* < 0.01 vs. control group without hypoxia 98% ± 1.7%). Extending the hypoxia time to 6 h did not significantly change the cell survival rate (31% ± 4.1%, *p* > 0.05 vs. 3 h hypoxia). Therefore, 3 h was identified as the hypoxia duration for the subsequent experiments. A median concentration (100 nM) was selected for testing DEX preconditioning for different durations (30 min, 1 h, 2 h, and 3 h) before hypoxia exposure. The MTT assay helped to confirm that the best and shortest DEX preincubation time was 1 h. The cell survival rate was 85% (*p* < 0.01 vs. the nonincubated Group). Reoxygenation for 1 h was chosen for all of the in vitro experiments.

**Figure 7:**
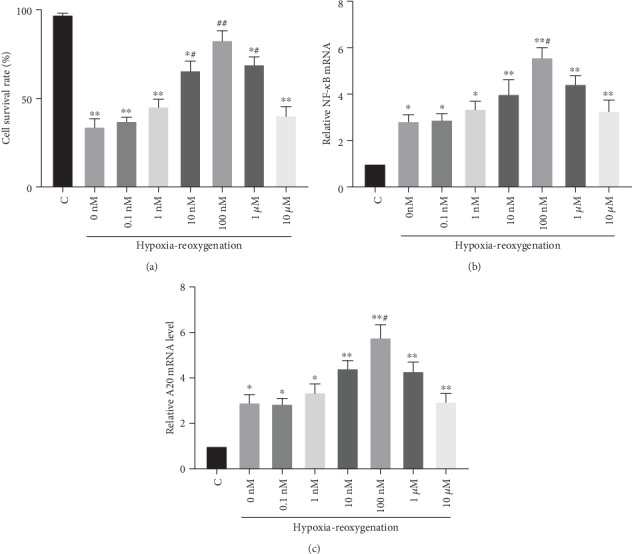
Effects of dexmedetomidine (DEX) concentrations on the survival rate and NF-*κ*B and A20 mRNA levels of human renal proximal tubular epithelial cells after hypoxia and reoxygenation. (a) Call survival rate. (b) NF-*κ*B mRNA. (c) A20 mRNA. The data are the mean ± standard deviation (SD). ^∗^*p* < 0.05, ^∗∗^*p* < 0.01 vs. C; ^#^*p* < 0.05, ^##^*p* < 0.01 vs. 0 nM DEX.

After DEX preconditioning at different concentrations (0 nM, 0.1 nM, 1 nM, 10 nM, 100 nM, 1 *μ*M, or 10 *μ*M) for 1 h, the HK-2 cells were subjected to hypoxia–reoxygenation, and cell viability was compared. Within a range of 0 to 100 nM, cell survival was positively correlated with the DEX concentration. It increased from 36% ± 6% (0 nM) to 77% ± 9% (100 nM). When the concentration of DEX was increased to 1 *μ*M, cell viability did not improve further (*p* > 0.05 vs. the 100 nM group). DEX preincubation at 10 *μ*M even led to decreases in cell viability (*p* < 0.05 vs. the 100 nM group). The changes in cell viability were positively correlated with the variations in NF-*κ*B and A20 (Figure 7).

## 5. Discussion

The study yielded two new findings. First, unlike the methylprednisolone application, DEX preconditioning exhibited a biphasic change of NF-*κ*B (activated NF-*κ*B within 3 h of IRI and inhibited NF-*κ*B 6 h after IRI) in the rat renal IRI models that were given 25 *μ*g/kg DEX i.p. It was accompanied by the early and persistent upregulation of A20 and early upregulation of TNF-*α* and significant inhibition in the later period. Second, in vitro, DEX also showed a biphasic cellular protection that was positively concentration-dependent within the range of 0 to100 nM (clinical doses) but negatively correlated with the concentration when it was greater than 1 *μ*M. The changes in the NF-*κ*B and A20 mRNA levels were consistent with the renoprotective ability of DEX. Thus, DEX preconditioning protected the rats from renal IRI via the fine regulation of NF-*κ*B signaling.

The unexpected findings that DEX preconditioning (25 *μ*g/kg i.p.) led to biphasic change of NF-*κ*B p65 signaling are different from those of previous studies. The following might be the reasons. First, there were differences in the observation time points in this study and previous studies [22, 30]. Few studies have observed changes in NF-*κ*B signaling in early reperfusion (especially within 3 h). Second, the differences in the experimental animals, organs, and doses and modes of DEX administration (i.p. or i.v., pre- or postconditioning) led to the variations in *α*2-AR agonism, *α*2-AR expression levels, and, eventually, changes in NF-*κ*B [22, 30–32].

Whether NF-*κ*B activation is an indication of organ damage or protection should not be concluded as merely being either good or bad. Indeed, the NF-*κ*B signaling system provides protection from extraneous challenges. Unfortunately, the protection afforded by NF-*κ*B-induced gene expression was not without a cost: the induction of local and systemic inflammation. The reason was that the downstream cytokines of NF-*κ*B included not only anti-inflammatory molecules (e.g., A20 and CYLD) but also proinflammatory molecules (e.g., chemokines, cytokines, and adhesion molecules) [33–35].

The systemic inhibition of NF-*κ*B by various methods has been found to ameliorate IRI. However, there are two aspects to the activation of NF-*κ*B: damage and protection. In ischemic pre- or postconditioning and drug preconditioning, NF-*κ*B is also responsible for tissue protection against IRI [36–42]. Specifically, the severity and duration of ischemia determine the outcomes for IRI [15]. Mild or brief ischemia causes the moderate activation of NF-*κ*B and promotes the formation of cytoprotective complexes. Severe or long-term ischemia leads to the strong activation of NF-*κ*B; the release of a large number of inflammatory factors; the recognition of Toll-like receptor (TLR), monocytes, or macrophages infiltration; and, eventually, the production of IRI.

In addition, an interesting result was that TNF-*α* initially increased within 3 h of DEX preconditioning and decreased significantly after 6 h. In this study, the early temporary increase of NF-*κ*B and TNF-*α* played a protective role. This is indicative of their possible involvement in the balance of inflammation. The mechanism was similar to that in ischemic preconditioning [38, 43]. Thus, it is possible that the maintenance of moderate NF-*κ*B activity in the early stage of inflammation or the inhibition of NF-*κ*B activation in the case of out-of-control inflammation could be both effective and noncontradictory.

In the first part of the present study, the effects of methylprednisolone and DEX were compared. Both methylprednisolone and DEX provided renoprotective effects during renal IRI even though they produced opposite changes in NF-*κ*B activation and TNF-*α* and A20 protein expression. This again demonstrated the duality of the physiological role of NF-*κ*B.

The findings provide an indication of a likely more elaborate but not well-understood underlying mechanism in DEX. The findings were interpreted in terms of the relationship between the GTP protein-coupled receptors and NF-*κ*B. G protein-coupled receptors (GPCRs) are widely distributed in the human body. They play an active role in regulating transcription and a variety of cellular functions. GPCRs use several possibly nonclassic pathways to activate NF-*κ*B [23, 24]. The aberrant regulation of the GPCR or NF-*κ*B signaling axis leads to the development of many diseases, such as cancer, inflammation, and autoimmunity. The *α*2-ARs are classic GPCR proteins. The inhibition of AC through G_i_ is the main signal transduction mechanism in all *α*2-AR subtypes [24]. On the one hand, it inhibits the AC-cAMP-PKA pathway directly through the G_*α*i_ proteins and suppresses the phosphorylation of the cAMP response element-binding protein (CREB) [35, 44]. On the other hand, the G_*β*_ and G_*γ*_ subunits dissociate from G_*α*i_ and activate the phosphatidylinositol 3-kinase- (PI3K-) protein kinase B (AKT) pathway, phospholipase C, extracellular regulated protein kinases (ERK), and p38 mitogen-activated protein kinase (p38MAPK) [23, 44–47]. Both pathways eventually lead to the activation of NF-*κ*B [23]. Therefore, the activation of NF-*κ*B, even temporarily, should be a reasonable result of DEX's excitation of the *α*2-ARs.

More interesting, the findings on the possible protective or damaging effects of *α*2-AR agonism are inconclusive [26, 27]. One study found that the application of DEX after myocardial infarction increased myocardial injury [48]. Gu et al. suggested that the organ-protective effects of DEX postconditioning are less than those of preconditioning [32]. Bunte et al. found that postconditioning with DEX was concentration-dependent in the range of 0.3 to 3 nM. Concentrations greater than 3 nM did not further enhance this cardioprotective effect [49]. It seems that there are multiple, and sometimes paradoxical, relationships between *α*2-AR agonism and organ protection. We speculate that different doses and timing of DEX treatment may be responsible for this. Further study should be made to get a better understanding.

Several previous studies have reported on the biphasic responses of AC or cAMP to *α*2-AR agonism during inflammation [24, 26, 50–52]. The findings indicated that *α*2-AR has inhibitory and stimulatory components. First, different *α*2-AR subtype agonists couple with G_i_ to inhibit AC to the same extent. However, if given high concentrations of agonist or high receptor expression, *α*2-ARs couple both physically and functionally with G_s_ and activate AC with a clear ranked order of *α*2A > *α*2B > *α*2C. Thus, with the gradual increase in the agonist concentrations, AC is first inhibited and then stimulated, and vice versa. Therefore, it is possible that the temporary activation of NF-*κ*B in the present study might be associated with the effects of DEX on AC. The reason is the changes in *α*2-AR expression accompanied by the inflammation caused by IRI or the changes in the plasma concentrations over time. A review of the literature indicates that this is the first evidence of the specific actions between DEX and NF-*κ*B.

Second, another new in vitro finding was the concentration-dependent biphasic effects of DEX on cell viability. Within 0 to 100 nM, cell viability, NF-*κ*B mRNA, and A20 mRNA were positively correlated with the DEX concentrations. When it exceeded 1 *μ*M, the NF-*κ*B mRNA, the A20 mRNA levels and cell viability began to decrease. Coincidentally, Lai et al. [42] found that with increases in DEX concentrations from 10 nM to 100 *μ*M, there were biphasic increases and then decreases in inducible nitric oxide synthase (iNOS), cyclooxygenase-2 (COX-2), TNF-*α*, IL-1*β*, and IL-6. Nishina et al. [53] noted that DEX caused neutrophil apoptosis when the clinically relevant dose was exceeded.

The optimal DEX concentration observed in both the present study and Lai et al.'s study was in the range of 10 to 100 nM [42], and this was consistent with the clinical dose range. Fraser et al. also found that at low concentrations (1–100 nM), epinephrine attenuated forskolin-stimulated cAMP accumulation in a receptor density-dependent manner and increased the cAMP levels at concentrations above 100 nM [50]. Therefore, all the results indicate that it is possible that DEX might be simultaneously coupled with G_s_ and G_i_ when preconditioned in the renal IRI in the in vivo and in vitro models. This results in time- and concentration-dependent biphasic changes in NF-*κ*B signaling.

In recent years, A20 has been found to have a protective effect in renal IRI. This occurs through the control of endogenous injury and the maintenance of the balance between pro- and anti-inflammatory responses [54–56]. It has been predicted that A20 will be one of the targets in the prevention and treatment of AKI and chronic kidney disease. In the in vivo phase of the present study, DEX preconditioning was found to continuously upregulate A20 expression in vitro and during perfusion. The A20 mRNA levels under the different concentrations of DEX were consistent with the changes in the cell survival rate. This confirms the importance of A20 in the protection of DEX. DEX's effects on non-IRI rats were observed. In addition, the mild upregulation of A20 was found not to be harmful. Whether A20 is the key to the protective effects of DEX need further study.

In sum, the results of this study indicate that DEX preconditioning does provide renal protection; however, the timing and dosage range might need to be carefully considered. Given that DEX could increase NF-*κ*B, it could cause organ damage and even increase the likelihood of cancer recurrence and metastasis [57, 58]. Further research is required to determine the *α*2-AR subtype responsible for the changes in NF-*κ*B signaling; the renoprotection of DEX postconditioning; and the role of G proteins, NF-*κ*B, and A20 in the process.

## Figures and Tables

**Table 1:** Urea nitrogen and creatinine levels at 24 h after ischemia–reperfusion injury.

	S	I	D+I	A+D+I	D	M+I
Cr(*μ*mol/L)	27.6 ± 2.2	253.5±34.1^∗∗^	30.8 ± 13^##^	247.5±23.7^∗∗^	21.5 ± 4.2^##^	45.6 ± 18.1^∗^^##^
BUN (mmol/L)	6.1 ± 1.1	47.9±5.0^∗∗^	10.2 ± 2.4^##^	51.7±7.5^∗∗^	6.4 ± 1.4^##^	15.5 ± 2.3^∗^^##^

The data are the mean ± standard deviation (SD; *n* = 5). Comparison with Group S, ^∗^*p* < 0.05, ^∗∗^*p* < 0.01; comparison with Group I, ^#^*p* < 0.05, ^##^*p* < 0.01.

## Data Availability

The data used to support the findings of this study are available from the corresponding author upon request.
